# The angiogenic properties of human amniotic membrane stem cells are enhanced in gestational diabetes and associate with fetal adiposity

**DOI:** 10.1186/s13287-021-02678-y

**Published:** 2021-12-20

**Authors:** Sergiy Klid, Francisco Algaba-Chueca, Elsa Maymó-Masip, Albert Guarque, Mónica Ballesteros, Cristina Diaz-Perdigones, Cristina Gutierrez, Joan Vendrell, Ana Megía, Sonia Fernández-Veledo

**Affiliations:** 1 Rovira i Virgili University, Tarragona, Spain; 2 Department of Endocrinology and Nutrition, Research Unit, University Hospital of Tarragona Joan XXIII-Institut d´Investigació Sanitària Pere Virgili (IISPV), Tarragona, Spain; 3 CIBER de Diabetes y Enfermedades Metabólicas Asociadas (CIBERDEM), Instituto de Salud Carlos III, Madrid, Spain; 4 Department of Obstetrics and Gynecology, University Hospital of Tarragona Joan XXIII, Tarragona, Spain

**Keywords:** Human amniotic stem cells, Gestational diabetes, Angiogenesis, Neonatal adiposity, Cord blood insulin, PAI-1

## Abstract

**Background:**

An environment of gestational diabetes mellitus (GDM) can modify the phenotype of stem cell populations differentially according to their placental localization, which can be useful to study the consequences for the fetus. We sought to explore the effect of intrauterine GDM exposure on the angiogenic properties of human amniotic membrane stem cells (hAMSCs).

**Methods:**

We comprehensively characterized the angiogenic phenotype of hAMSCs isolated from 14 patients with GDM and 14 controls with normal glucose tolerance (NGT). Maternal and fetal parameters were also recorded. Hyperglycemia, hyperinsulinemia and palmitic acid were used to in vitro mimic a GDM-like pathology. Pharmacological and genetic inhibition of protein function was used to investigate the molecular pathways underlying the angiogenic properties of hAMSCs isolated from women with GDM.

**Results:**

Capillary tube formation assays revealed that GDM-hAMSCs produced a significantly higher number of nodes (*P* = 0.004), junctions (*P* = 0.002) and meshes (*P* < 0.001) than equivalent NGT-hAMSCs, concomitant with an increase in the gene/protein expression of FGFR2, TGFBR1, SERPINE1 and VEGFA. These latter changes were recapitulated in NGT-hAMSCs exposed to GDM-like conditions. Inhibition of the protein product of *SERPINE1* (plasminogen activator inhibitor 1, PAI-1) suppressed the angiogenic properties of GDM-hAMSCs. Correlation analyses revealed that cord blood insulin levels in offspring strongly correlated with the number of nodes (*r* = 0.860; *P* = 0.001), junctions (*r* = 0.853; *P* = 0.002) and meshes (*r* = 0.816; *P* = 0.004) in tube formation assays. Finally, *FGFR2* levels correlated positively with placental weight (*r* = 0.586; *P* = 0.028) and neonatal adiposity (*r* = 0.496; *P* = 0.014).

**Conclusions:**

GDM exposure contributes to the angiogenic abilities of hAMSCs, which are further related to increased cord blood insulin and fetal adiposity. PAI-1 emerges as a potential key player of GDM-induced angiogenesis.

## Background

The maternal environment is a major factor in fetal growth and development. Several lines of evidence show that prenatal exposure to nutritional and metabolic stress is associated with fetal reprogramming, a concept that considers specific environmental events occurring during pregnancy as a cause of adverse effects long after birth [1, 2]. For example, the hyperglycemic and hyperlipidemic environment found in gestational diabetes (GDM)—one of the most common complications in pregnancy—increases the risk of cardiovascular and metabolic disorders such as obesity and type 2 diabetes later in life [3–6].

Functional integrity of the placenta, the natural interface between mother and fetus, is crucial to protect the fetus from fluctuations in maternal metabolic and energy status [7]. While the mechanisms underlying fetal programming remain elusive, a growing body of evidence points to the metabolic imprint of fetal precursor cells in the placenta and/or umbilical cord as a potential driver of negative health outcomes in offspring. Along this line, several studies have demonstrated that cord- and/or placental-derived mesenchymal stem cells (MSCs) isolated from women with GDM exhibit unique proliferative [8, 9], immunomodulatory [8] and cell differentiation [10, 11] properties as compared with equivalent cells from healthy pregnancies. It has been also described that GDM alters the transcriptional profile [12] and mitochondrial activity [11, 13] of these precursor cell populations. In this context, we recently demonstrated that GDM disturbs the multilineage differentiation potential and immunomodulatory properties of human amniotic MSCs (hAMSCs) [14], which are located in a privileged region in close contact with the amniotic fluid and the fetus. We also found that the inflammatory status of hAMSCs was related not only to maternal metabolic cues, but also to infant clinical and metabolic status [14], indicating that hAMSCs might be a powerful tool for the indirect study of fetal cells in the context of hyperglycemia and insulin resistance.

While it might seem contradictory, fetal precursors from the amniotic membrane—one of the few avascular tissues—secrete significant amounts of angiogenic factors [15, 16] and have strong angiogenic potential [16–19]. Pre-clinical research exploiting this phenomenon by using hAMSCs as a cell-based therapy has produced encouraging results in several disease models [18, 20–23]. While it remains unclear what role hAMSCs with pronounced vascular properties have in normal physiology, they might reflect specific characteristics of the offspring (e.g., vascular system), as has been proposed for endothelial cells in the umbilical cord [24]. Indeed, umbilical cord-derived primary cells from offspring of mothers with GDM have compromised angiogenic capacity measured as in vitro capillary tube formation activity, which is in agreement with the evident differences in gene expression of pathways related to angiogenesis and vascular development [25]. In the present study, we sought to determine the effect of intrauterine exposure to GDM on the angiogenic properties of hAMSCs, and whether their vascular characteristics are related to maternal and infant metabolic status.

## Methods

### Study population

Twenty-seven pregnant women (14 with GDM and 14 with NGT, acting as controls) scheduled for cesarean delivery, were recruited at the Hospital Universitari de Tarragona Joan XXIII over 32 months. Pregnant women with either pre-existing type 1 or type 2 diabetes, inflammatory or chronic diseases, or taking drugs known to affect carbohydrate metabolism, were excluded from the study cohort. All participants were screened for GDM at 24–28 weeks of gestation using a two-step approximation according to the standards of the Spanish Group on Diabetes and Pregnancy guidelines, which followed the National Data Group Criteria [26, 27]. Mothers diagnosed with GDM followed an individualized diet with 40% of carbohydrates and had to self-monitor blood glucose 6 times daily (fasting and 1-h postprandial). Insulin therapy was initiated when fasting glucose was 95 mg/dL or higher and/or 1-h postprandial values were above 140 mg/dL. The study was performed according to the tenets of the Helsinki Declaration. Ethical approval was obtained by the Institut d’Investigació Sanitaria Pere Virgili Research Ethics Board (Ref: 133/2018), and all participants signed a written informed consent before entering the study.

### Clinical and demographic data

Gestational age was confirmed by a routine ultrasonographic examination scheduled before week 20 of gestation. Maternal pre-pregnancy weight and height were annotated by self-report at the first prenatal visit. Pre-pregnancy body mass index (BMI) and gestational weight gain (GWG) were calculated using the following formulas: pre-pregnancy BMI = pre-pregnancy weight (kg)/(height (m))^2^ and GWG = final weight—pre-pregnancy weight. Gestational timing of delivery was based mainly on obstetric indications.

Offspring body and placenta weight were annotated after parturition, and neonatal tricipital, subscapular and suprailiac skinfold thickness were measured using a Holtain skinfold caliper (Chasmors Ltd., London, UK) within 48 h.

### Sample collecting and processing

Maternal blood was drawn in the morning, after at least 8 h of fasting and just prior to the cesarean section; umbilical cord blood was obtained immediately after delivery. Serum and plasma were processed and stored at -80ºC until analysis. Full-term placentas were collected after delivery and processed under sterile conditions within one hour. The amniotic membrane was manually peeled from the underlying chorionic membrane and washed with phosphate-buffered saline (PBS) containing a 1% antibiotic/antimycotic solution.

### Laboratory measurements

Glucose, cholesterol and triglyceride levels were determined with ADVIA 1800 and 2400 (Siemens AG, Munich, Germany) autoanalyzer platforms following standard enzymatic methods. An immunoassay on the Centaur XP platform (Siemens AG) was assessed to determine fasting insulin.

### Isolation and culture of human amniotic membrane mesenchymal stem cells

To isolate hAMSCs, samples of amniotic membrane were minced and subjected to 30-min digestion with 0.25% trypsin–EDTA solution, followed by a 90-min, second digestion with collagenase type IV (Gibco, Carlsbad, CA) dissolved in complete medium containing Dulbecco’s modified Eagle’s medium (DMEM)/F12 (1:1, 1 × , HyClone, Logan, UT), 10% fetal bovine serum (FBS, HyClone) and 1% antibiotic/antimycotic solution (Gibco). After centrifuging and washing, the pellet was resuspended in the aforementioned medium. Primary cultures of hAMSCs at passage 0 were grown to 80–90% confluence at 37 °C in 5% CO_2_, with a medium change 1 day after seeding. Non-adherent cells were removed by rinsing twice with PBS. After 4 days, hAMSCs were recovered using 0.25% trypsin–EDTA and seeded in 75-cm^2^ flasks and cultured in DMEM/F12, with 10% FBS and 1% antibiotic/antimycotic solution, changing the medium every 2 days. hAMSCs were grown to passage 3 using the same culture medium. To mimic the GDM-like environment for capillary-tube formation assays, control hAMSCs were serum-deprived in DMEM/F12 supplemented with 0.2% BSA (Sigma-Aldrich, St. Louis, MO) at least 2 h prior to a 24-h (for tube formation analysis) or 48-h stimulation (for protein analysis) with glucose, insulin and palmitic acid at a final concentration of 30 mM, 100 nM and 50 µM, respectively.

### Flow cytometry analysis

Surface markers of the mesenchymal lineage were determined and analyzed by flow cytometry (FACS Aria III; BD Biosciences, San Jose, CA) meeting the minimum criteria defined by the International Society of Cell Therapy [28]. Briefly, hAMSCs (1 × 10^5^) were incubated with a panel of primary antibodies (BD Pharmingen, San Diego, CA) as described [14]. Data analysis was performed using FACSDiva software (BD Biosciences). Cells were positive for the surface markers CD90 (96.95 ± 2.315), CD73 (93.73 ± 4.885) and CD105 (90 ± 6.205) and negative for CD45 (0.02 ± 0.05), CD34 (0.12 ± 0.17), CD31 (0.31 ± 0.33) and CD14 (0.34 ± 0.5).

### Gene expression analysis

Total RNA was extracted using TRIzol® Reagent (Invitrogen), and its quality was assessed by the 260/280 nm optical density ratio. Two micrograms of total RNA was transcribed into cDNA with random primers using a dNTP Mix (100 mM), MultiScribe Reverse Transcriptase (50 U/μL) and RNase Inhibitors with the High-Capacity cDNA Reverse Transcription Kit (Applied Biosystems, Foster City, CA). Gene expression was evaluated by quantitative reverse transcription real-time PCR (RT-qPCR) on a 7900HT Fast Real-Time PCR System (Applied Biosystems) using a predesigned TaqMan Low Density Array (Applied Biosystems), comprising the following genes: *ANG* (Hs00265741_s1), *ANGPT1* (Hs00919202_m1), *ANGPT2* (Hs00169867_m1), *ANGPT4* (Hs00907074_m1), *ANGPTL4* (Hs01101123_g1), *CCL2* (Hs00234140_m1), *IL8* (Hs00174103_m1), *FGF-2* (Hs00266645_m1), *FGFR2* (Hs01552918_m1), *IGF1* (Hs01547656_m1), *IGF1R* (Hs00609566_m1), *IL1β* (Hs01555410_m1), *MMP2* (Hs01548727_m1), *MMP9* (Hs00957562_m1), *PDGFB* (Hs00966522_m1), *PDGFRA* (Hs00998018_m1), *PDGFRB* (Hs01019589_m1), *SERPINE1* (Hs00167155_m1), *TGFβ1* (Hs00998133_m1), TGFβR1 (Hs00610320_m1), TNF (Hs00174128_m1), VEGFA (Hs00900055_m1), *VEGFR1* (Hs01052961_m1) and *VEGFR2* (Hs00911700_m1). Gene expression values were calculated using the comparative Ct method (2 − ΔΔCt) and normalized to the expression of the housekeeping gene *18S* (Hs03928985_g1).

### Protein expression analysis

Cells were lysed and homogenized in M-PER buffer (Thermo Scientific, Waltham, MA) containing a protease and phosphatase inhibitor cocktail; protein concentration was determined with the BCA Protein Assay Kit (both from Pierce Biotechnology, Rockford, IL). Equal amounts of total protein were separated using SDS-PAGE electrophoresis, transferred to Immobilon-*P* PVDF Membranes (Merck Millipore, Burlington, MA) and blocked in 5% non-fat milk in T-TBS. Immunoblotting was performed using polyclonal antibodies against FGFR2 (F6796, Sigma-Aldrich), TGFBR1 (SAB1300113, Sigma-Aldrich), PAI-1 (612,024, BD Transduction Laboratories, Franklin Lakes, NJ) and a monoclonal antibody against VEGFA (19,003–1-AP, Proteintech Group, Manchester, UK). Anti-β-actin (Sigma-Aldrich) was employed as a loading control. Western blot signals were visualized using the Immobilon ECL Ultra Western HRP Substrate (Merck Millipore) and captured on an iBright CL1000 Imaging System equipped with iBright Analysis Software (Invitrogen, Carlsbad, CA).

### Small molecule inhibitors

Alofanib, SB-431542 and TM5275 sodium inhibitors (MedChemExpress LLC, Monmouth Junction, NJ) were used as small molecule inhibitors of FGFR2, TGFBR1 and PAI-1, respectively. Inhibitors (purchased lyophilized) were dissolved in dimethyl sulfoxide (DMSO) at a stock concentration of 1, 5 and 10 mM, respectively, and were stored at -80 °C. Serum-deprived (2 h) hAMSCs were treated for 3 h with alofanib (1–5 μM), SB-431542 (10–50 μM) or TM5275 sodium (50–100 μM) diluted in DMEM/F12 with 0.2% BSA prior to the tube formation assay. Inhibiting concentrations were maintained in the medium when plating on the gel matrix. Controls were treated with equivalent amounts of DMSO.

### siRNA-mediated knockdown

RNA interference-mediated gene silencing was used to target VEGFA protein function. hAMSCs were plated at 7.5 × 10^4^ cells per well in a 12-well plate and allowed to adhere overnight. Prior to transfection, cells were serum-deprived for 2 h in Opti-MEM I Reduced Serum Medium (Gibco). ON-TARGETplus VEGFA siRNA and ON-TARGETplus Non-targeting Control Pool (Horizon Discovery Ltd., Waterbeach, UK) were diluted in Optimem-MEM to a concentration of 100 nM. The same reduced serum medium was used to dilute the transfection reagent Lipofectamine RNAiMAX (Invitrogen). Diluted RNAi duplexes and Lipofectamine RNAiMAX were combined, reducing the siRNA final concentration to 50 nM, and 500 µL of transfection mixture was added to each corresponding well. Cells were incubated for 48 h at 37ºC and 5% CO_2_. The transfection medium was diluted 1:2 after 6 h and was completely replaced with complete growth medium at 24 h post-transfection. Successful siRNA delivery and *VEGFA* gene knockdown efficiency were verified with RT-qPCR.

### Tube formation assay

Analysis of capillary formation was performed using an extracellular gel matrix from Engelbreth-Holm-Swarm mouse sarcoma cells (Sigma-Aldrich). In total, 60 μL of gel matrix solution was applied to each well of a 96-well plate and incubated for 30 min at 37 °C. hAMSCs (1.2 × 10^4^) were suspended in 100 μL of DMEM/F12 with 0.2% BSA, plated onto the gel matrix in triplicate and incubated at 37ºC. After 4 h of incubation, at least 5 fields were randomly photographed using a ZEISS Primovert microscope (ZEISS, Oberkochen, Germany). The number of extremities, nodes, junctions and meshes of each sample was measured using Angiogenesis Analyzer for ImageJ (National Institutes of Health, Bethesda, Maryland), as described [29].

### Statistical analysis

Statistical calculations and visualizations were performed using GraphPad software version 8.0 (GraphPad Software Inc., San Diego, CA). For clinical, metabolic and gene expression data integration we used SPSS software version 20.0 (IBM, Armonk, NY). Clinical data are expressed as mean ± SD for quantitative variables and as number (percentages) for categorical variables. In vitro data are shown as mean ± SEM. The one-sample Kolmogorov–Smirnov test was used to verify normal distribution of the data. Statistical significance was tested by Student’s t test (two-tailed, 95% confidence interval) or Mann–Whitney U test, as required. Pearson’s correlation coefficient analysis (two-tailed, 95% confidence interval) was used to examine the relationship between gene expression and clinical and metabolic parameters. A *P*-value < 0.05 was considered statistically significant in all analyses.

## Results

### Clinical and analytical characteristics of the cohort

Clinical, anthropometric and analytical data from mothers in pregnancy and their offspring are shown in Table 1. Pregestational BMI was significantly higher (*P* = 0.01) in mothers with GDM (*N* = 14) than in peers with normal glucose tolerance (NGT) (*N* = 14), but no other differences were found for maternal metabolic parameters. Likewise, no significant differences were found for offspring, placental and neonatal anthropometrical measurements or for biochemical characteristics between the GDM and NGT groups.

**Table 1:** Clinical and analytical characteristics of the cohort

	NGT (*N* = 14)	GDM (*N* = 14)	*P*-value
*Maternal clinical and analytical characteristics*			
Maternal age (years)	33.86 ± 6.97	36.57 ± 3.65	0.208
Pregestational BMI (kg/m^2^)	25.27 ± 3.29	30.33 ± 5.93	0.010
Gestational weight gain (kg)	12.37 ± 4.51	8.21 ± 6.10	0.051
Nulliparous, *n* (%)	6 (43)	2 (14)	0.092
Glucose (mg/dL)	4.88 ± 1.94	4.65 ± 1.03	0.704
*Offspring clinical and analytical characteristics*			
Gestational week delivery (weeks)	37.71 ± 0.73	38.07 ± 0.47	0.136
Birth weight (g)	3.265.36 ± 330.32	3.318.93 ± 379.89	0.694
Placental weight (g)	632.86 ± 47.51	675.00 ± 193.24	0.586
Sum skinfolds (mm)	12.47 ± 1.09	12.67 ± 2.20	0.773
Cb glucose (mg/dL)	3.57 ± 0.58	3.78 ± 0.78	0.437
Cb insulin mUI/L	33.89 ± 23.36	58.75 ± 50.83	0.295
Cb C peptide ng/dL	0.24 ± 0.06	0.27 ± 0.17	0.548

Data expressed as mean ± SD for quantitative variables following normal distribution, qualitative variables are expressed as *n* (%). Differences between quantitative variables were assessed by Student’s t test or the Mann–Whitney *U* test, as required. Differences between qualitative variables were assessed by the Chi-square test

The table should appear in the “Clinical and analytical characteristics of the cohort” subsection, inside the “Results”

*NGT* normal glucose tolerance, *GDM* gestational diabetes mellitus, *BMI* body mass index, *Cb* Cord blood

### GDM environment enhances the angiogenic capacity of hAMSCs

**Fig. 1:**
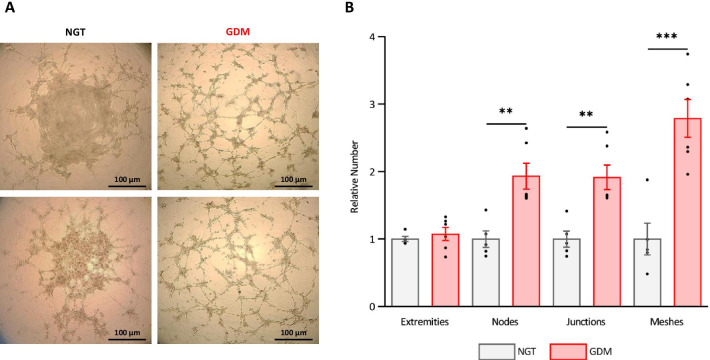
GDM environment enhances the angiogenic capacity of hAMSCs. **A** Visual representation of tube formation assay of NGT-hAMSCs and GDM-hAMSCs. Two randomly imaged representative fields of each group are represented. Scale bars = 100 µm. **B** Quantitative data represent differences in the number of extremities, nodes, junctions and meshes between NGT- and GDM-hAMSCs (*n* = 11; 5 NGT and 6 GDM). Data are normalized to the mean of the controls of each experiment and are shown as mean ± SEM. Differences assessed by Student’s *t* test. **P < 0.01; ***P < 0.001

hAMSCs were isolated from the amniotic membrane of women in both groups after delivery. To test the angiogenic potential of hAMSCs, we employed a functional tube formation assay that evaluates their ability to form capillary-like structures in an extracellular matrix (ECM). Randomly photographed representative fields of the angiogenic networks revealed markedly higher numbers of branching points and longer tube lengths from GDM-hAMSCs than from NGT-hAMSCs (Fig. 1a). Accordingly, the extent of tube formation was significantly greater in GDM-hAMSCs than in NGT-hAMSCs, measured as the number of capillary nodes (*P* = 0.004), junctions (*P* = 0.002) and meshes (*P* < 0.001), whereas the number of extremities was similar (*P* = 0.520) (Fig. 1b).

### GDM-hAMSCs display an enhanced pro-angiogenic expression profile

**Fig. 2:**
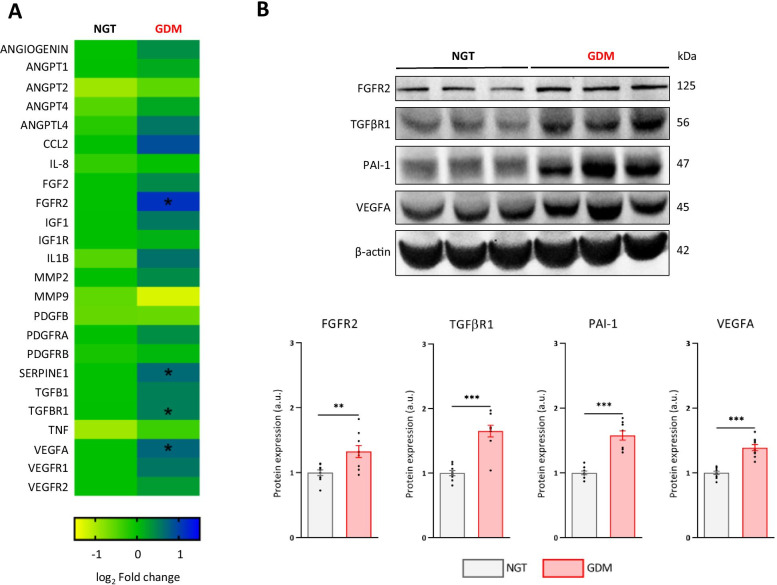
GDM-hAMSCs exhibit a specific pro-angiogenic expression profile. **A** Heatmap comparing angiogenic gene expression between the GDM and NGT groups (*N* = 14 in each). Data are represented as log_2_ (fold change). Differences assessed by Mann–Whitney U test. **B** Western blot analysis of FGFR2, TGFBR1, PAI-1 and VEGFA protein expression. Representative image and densitometry analysis (arbitrary units, *N* = 9 each group). Data are normalized to the mean of the controls of each experiment and are shown as mean ± SEM. Differences assessed by Student’s *t* test. *P < 0.05; **P < 0.01; ***P < 0.001

After bibliographic screening, we selected 24 genes as angiogenic markers and performed expression profiling using a predesigned TaqMan Low Density Array (see Methods). Comparative analysis revealed that the expression of fibroblast growth factor receptor 2 (*FGFR2*; *P* = 0.017), serpin family E member 1 (*SERPINE1*; *P* = 0.019), transforming growth factor beta receptor 1 (*TGFBR1*; *P* = 0.038) and vascular endothelial growth factor A (*VEGFA*; *P* = 0.033) was significantly greater in GDM-hAMSCs than in NGT-hAMSCs (Fig. 2a), which was also confirmed at the protein level by western blotting (Fig. 2b).

### A GDM-like environment induces angiogenic activity in healthy hAMSCs

**Fig. 3:**
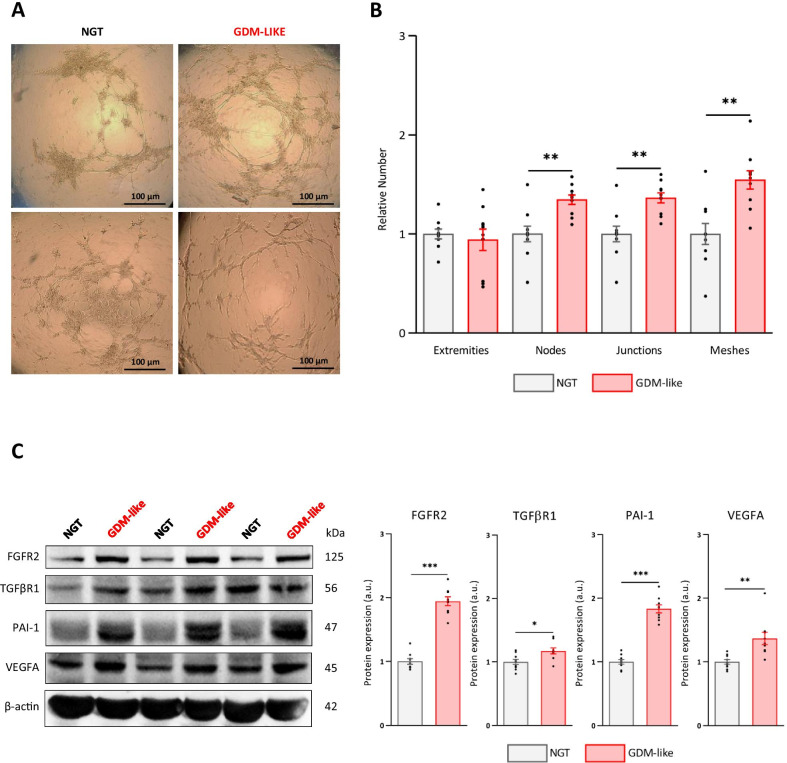
GDM-like milieu confers a pro-angiogenic profile on NGT-hAMSCs. NGT-hAMSCs were cultured in the presence of 30 mM glucose, 100 nM insulin and 50 µM palmitic acid. **A** Visual representation of tube formation assay of NGT-hAMSCs and GDM-like treated hAMSCs. Two representative fields of each condition are shown. Scale bars = 100 µm **B** Differences in the number of extremities, nodes, junctions and meshes between groups (*N* = 10 each group). **C** Protein expression analysis comparing angiogenic protein levels of FGFR2, TGFBR1, PAI-1 and VEGFA between groups. Representative image and densitometry analysis (arbitrary units, *N* = 9 each group). Data are normalized to the mean of the controls of each experiment and are shown as mean ± SEM. Differences assessed by Student’s *t* test. *P < 0.05; **P < 0.01; ***P < 0.001

To validate the angiogenic characteristics of GDM-hAMSCs, we attempted to mimic a GDM-like environment in vitro by culturing NGT-hAMSCs under conditions of hyperglycemia, hyperinsulinemia and dyslipidemia (using the saturated fatty acid palmitic acid). Comparative analysis showed that NGT-hAMSCs cultured in a GDM-like environment were very similar to GDM-hAMSCs with respect to their angiogenic properties (Fig. 1a), including a markedly higher number of branching points and longer tube lengths in comparison with healthy NGT-hAMSCs (Fig. 3a). This effect was reflected in a significantly higher number of nodes (*P* = 0.002), junctions (*P* = 0.001) and meshes (*P* = 0.001), whereas the number of extremities remained similar (*P* = 0.626) (Fig. 3b). Likewise, western blotting analysis revealed that a GDM-like insult led to a significant increase in the expression of FGFR2, TGFBR1, plasminogen activator inhibitor 1 (PAI-1) and VEGFA in NGT-hAMSCs (Fig. 3c).

### PAI-1 has a key role in the GDM-related proangiogenic properties of hAMSCs

**Fig. 4:**
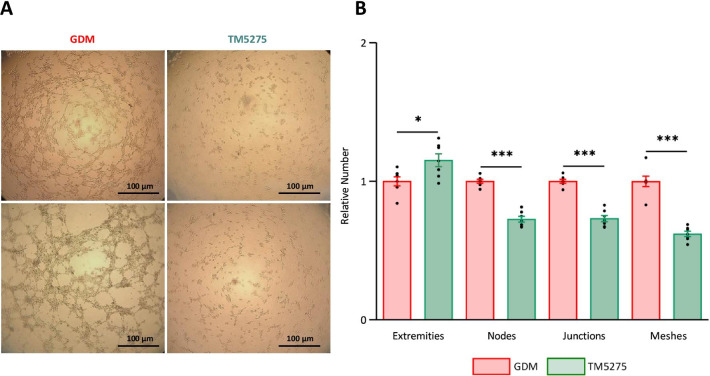
PAI-1 inhibition by TM5275 decreases the angiogenic properties of GDM-hAMSCs. GDM-hAMSCs were treated with 100 µM TM5275. **A** Visual representation of tube formation assay. Two representative fields of each condition are shown. Scale bars = 100 µm. **B** Quantitative data represent differences in the number of extremities, nodes, junctions and meshes between GDM- and TM5275-treated GDM-hAMSCs (*N* = 7 in each group). Data are normalized to the mean of the controls of each experiment and are shown as mean ± SEM. Differences assessed by Student’s t test. *P < 0.05; ***P < 0.001

To decipher the potential involvement of the proteins upregulated in GDM-hAMSCs, we followed two approaches: pharmacological inhibition of protein function for FGFR2, TGFBR1 and PAI-1, and siRNA-mediated knockdown for VEGFA. The ability to form vascular networks was assessed in all cases. Results showed that neither alofanib (1–5 µM) nor SB-431542 (10–50 µM), selective inhibitors of FGFR2 and TGFBR1, respectively, nor VEGFA siRNA-mediated knockdown (50 nM), altered the pro-angiogenic ability of GDM-hAMSCs, as no significant differences were found in any of the 4 parameters analyzed (extremities, nodes, junctions and meshes) compared with vehicle treatment (data not shown). By contrast, when we blocked PAI-1 function using the TM5275 sodium inhibitor (100 µM), we observed a notable disruption of the tubular network and a lower number of branching points, as compared with the non-treated group (Fig. 4a). This was reflected in a significant reduction in the number of capillary nodes (*P* < 0.001), junctions (*P* < 0.001) and meshes (*P* < 0.001) in GDM-hAMSCs treated with TM5275 compared with the control group (Fig. 4b).

### Angiogenic properties of fetal precursor cells are related to neonatal anthropometric and metabolic features

**Fig. 5:**
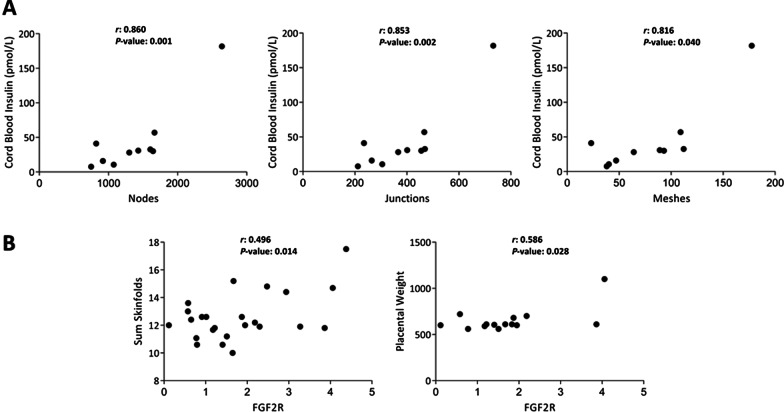
Functional angiogenic properties of hAMSCs are strongly associated with infant metabolic parameters (*N* = 11; 6 NGT and 5 GDM) (**A**), and their gene signature is related to placental weight (*N* = 14; 7 in each group) and neonatal adiposity parameters (*N* = 25; 13 NGT; 12 GDM) (**B**). Gene expression data are expressed as fold change, whereas functional parameters appear as numbers. Correlations were calculated using Pearson’s correlation coefficient

We next explored the potential relationship between maternal anthropometric and metabolic parameters and the angiogenic phenotype of hAMSCs, including whether their functional characteristics were associated with offspring clinical and analytical parameters. Correlation analysis of the entire cohort revealed that the levels of offspring cord blood insulin strongly correlated with the number of nodes (*r* = 0.860; *P* = 0.001), junctions (*r* = 0.853; *P* = 0.002) and meshes (*r* = 0.816; *P* = 0.004) in tube-forming assays (Fig. 5a). No correlations were found when we analyzed each group separately. In a similar analysis, we explored the relationship between the differentially expressed genes and clinical parameters. We found that *FGFR2* expression significantly correlated with the neonate sum of skinfolds (*r* = 0.496; *P* = 0.014) and with placental weight (*r* = 0.586; *P* = 0.028) (Fig. 5b). No correlations were found when analyzing each group separately.

## Discussion

The placenta is a rich reservoir of perinatal stem cells with recognized therapeutic potential for cell-based therapies due to their superior pro-angiogenic features, among other properties. At the same time, examination of fetal precursors can be useful to better understand pregnancy disorders and potential consequences for the fetus, as they can reflect disturbances in maternal homeostasis (e.g., GDM) [30]. The impact of GDM for placental angiogenesis is unclear, and to the best of our knowledge, no studies have examined how GDM might influence the angiogenic properties of MSCs from the amniotic membrane. Our analysis reveals that hAMSCs isolated from the placenta of mothers with GDM show an upregulation of genes involved in angiogenesis, including *TGFβR*1, *VEGFA*, *FGFR2* and *SERPINE1*, with the latter being a potential key player in angiogenesis regulation. Moreover, the expression of *FGFR2* was closely associated with neonatal adiposity and placental weight. The pro-angiogenic expression phenotype in hAMSCs from GDM mothers correlated with enhanced angiogenic capacities, which were additionally related to fetal insulin concentration. Overall, these data support the hypothesis that the in utero environment impacts the fetal phenotype, and hAMSCs should be considered an important factor in this interconnection.

The amniotic membrane is a thin, transparent and avascular membrane that envelopes the fetus and holds the amniotic fluid [31]. It contains two types of fetal stem cells: epithelial stem cells from the innermost layer of amnion, which directly contact with amniotic fluid and fetus, and hAMSCs scattered in the membrane [32]. hAMSCs are known to express and produce pro-angiogenic factors needed for neo-vascularization and angiogenesis [20], and also have the capacity to differentiate into endothelial cells [32, 33], although their ability to induce a stable vascular network has been questioned [19]. hAMSCs are also able to stimulate angiogenesis in stem cells derived from other tissues, such as human adipose tissue mesenchymal stem cells [34], and to form endothelial rings [35]. By contrast, the epithelial surface that sustains the epithelial stem cells seems to have an inhibitory effect on vessel formation, which may inhibit hAMSC differentiation into mature endothelial cells and maintain the avascularity of the amniotic membrane [31, 32, 36]. The impact of a GDM environment on placental angiogenesis is a contentious issue, with some authors finding defective vascular tree formation and altered angiogenesis in placentas from mothers with diabetes [37], and others reporting hypervascularization [38]. Placental chorionic mesenchymal stem cells were reported to have poor tube formation ability in GDM [39], concomitant with systemic downregulation of angiogenic genes [12]. Similarly, the GDM environment was found to disturb the migration, proliferation and tube formation capacities of human umbilical vein endothelial cells [40]. In contrast to these data, the present study reveals that hAMSCs from GDM placentas display superior angiogenic activity over healthy counterparts, which suggests that the impact of GDM on fetal precursors is likely cell-specific. Additionally, NGT-hAMSCs exposed to a GDM-like environment display GDM-hAMSCs features, with an increase in the expression of angiogenic proteins and in angiogenic potential. Indeed, it has been described that hyperglycemia and hyperinsulinemia—the two main characteristics of GDM—contribute to hypervascularization in fetoplacental endothelial cells [41] and in the trophoblast [42]. In this context, we found that cord blood insulin concentration, a surrogate marker of the fetal environment, was closely related to the angiogenic capacity of hAMSCs measured as nodes, junctions and meshes.

Angiogenesis is a highly regulated process that involves multiple cellular components and proteins including VEGF, FGF2, PAI-1 and TGFβ1 [38, 43, 44]. VEGF, an inducer of endothelial cell proliferation activation and migration, is one of the main angiogenic regulators, ensuring the adequate supply of nutrients and oxygen to the fetus [38]. The role VEGF and its receptors in placental angiogenesis in GDM are, however, poorly established [45, 46]. In accordance with the higher angiogenic activity in GDM-isolated hAMSCs, we found that the expression of *VEGFA* in hAMSCs of GDM mothers was elevated, suggesting a deregulation of the protein. Our data contrast with previous reports that observed a downregulation of VEGF in MSCs of the highly vascularized chorion [39]. These differences may be reconciled in the context of the stem cell niche, which is a key regulator of the properties of specific stem cell populations.

Other angiogenesis-related genes such as *TGFBR1*, *SERPINE1* and *FGFR2* were up-regulated in GDM-hAMSCs. The effects of TGFβ1 on endothelial cells seem to be concentration-dependent and can be enhanced or inhibited in the presence of other regulators [47], and hyperglycemia increases its activity [48, 49]. TGFβR1 signaling regulates *SERPINE1* (PAI-I) expression. PAI-1 has been associated with a high pro-angiogenic activity at physiological concentrations [50], and its up- or downregulation promotes or inhibits blood vessel formation, respectively [51, 52]. PAI-1 has been previously reported to be elevated in the serum of patients with GDM [43], and it is known to be upregulated by the pro-angiogenic placental growth factor gene [53]. In agreement with these data, we observed that TM5275, a PAI-1 selective pharmacological inhibitor, substantially attenuated the angiogenic capacity of GDM-hAMSCs. These findings suggest that some of these proteins, and specifically PAI-1, play a pivotal role in balancing the angiogenic process. It is well established that FGF2 signaling participates in the regulation of human placental artery endothelial cell proliferation and angiogenesis [54, 55]. Of note, *FGFR2* expression correlated significantly with neonatal sum of skinfolds and placental weight, which accords with a previous report showing a positive relationship between FGF2 and birth-weight and placental size in pregnancies complicated by diabetes [57]. This association might be mediated by DNA methylation of FGFR2 [55].

It is important to note that all placental material was obtained from a well-characterized cohort of mothers scheduled for cesarean delivery, to avoid any uncontrolled inflammatory stimuli linked to labor, which could affect the angiogenic phenotype of hAMSCs. Fasting maternal blood samples were obtained just prior to cesarean section, and GDM was well controlled, including insulin therapy when needed. This situation obviously limited our ability to investigate differences in metabolic parameters between the groups. Another limitation of our study is the relatively small sample size; however, it is substantially larger than other studies analyzing the plasticity of placental-derived mesenchymal stromal cells [9, 13, 14, 25]. Finally, we cannot discard the possibility that the stemness and angiogenic abilities of the hAMSCs were affected by cryopreservation and serial passaging [58], but as the same protocol was applied to GDM- and NGT-derived hAMSCs it should not affect our results.

## Conclusion

Our study demonstrates that the GDM environment enhances the angiogenic potential of hAMSCs, which is at least partly mediated by PAI-1, and linked to some neonatal metabolic markers. From a therapeutic perspective, the pro-angiogenic potential of GDM-hAMSCs points to these cells as good candidates for future cellular-based therapies aimed to improve dysfunctional angiogenesis.

## Data Availability

The datasets used and/or analyzed during the current study are available from the corresponding authors on reasonable request.

## Abbreviations

GDM: Gestational diabetes mellitus
NGT: Normal glucose tolerance
hAMSCs: Human amniotic membrane stem cells
NGT-hAMSCs: Normal glucose tolerance-derived human amniotic membrane stem cells
GDM-hAMSCs: Gestational diabetes mellitus-derived human amniotic membrane stem cells
BMI: Body mass index
GWG: Gestational weight gain
ECM: Extracellular matrix
dTNP: Deoxyribonucleotide triphosphate
cDNA: Complementary DNA
RT-qPCR: Quantitative reverse transcription real-time polymerase chain reaction
FGFR2: Fibroblast growth factor receptor 2
PAI-1: Plasminogen activator inhibitor 1
SERPINE1: Serpin family E member 1
TGFβR1: Transforming growth factor beta receptor 1
VEGFA: Vascular endothelial growth factor A
MSC: Mesenchymal stem cell
VEGF: Vascular endothelial growth factor
TGFβ1: Vascular transforming growth factor beta 1
