# Correction: The angiogenic properties of human amniotic membrane stem cells are enhanced in gestational diabetes and associate with fetal adiposity

**DOI:** 10.1186/s13287-023-03420-6

**Published:** 2023-07-27

**Authors:** Sergiy Klid, Francisco Algaba-Chueca, Elsa Maymó-Masip, Albert Guarque, Mónica Ballesteros, Cristina Diaz-Perdigones, Cristina Gutierrez, Joan Vendrell, Ana Megía, Sonia Fernández-Veledo

**Affiliations:** 1 Rovira I Virgili University, Tarragona, Spain; 2 Department of Endocrinology and Nutrition, Research Unit, University Hospital of Tarragona Joan XXIII-Institut d´Investigació Sanitària Pere Virgili (IISPV), c/ Dr. Mallafré Guasch, 4, 43007 Tarragona, Spain; 3 CIBER de Diabetes Y Enfermedades Metabólicas Asociadas (CIBERDEM), Instituto de Salud Carlos III, Madrid, Spain; 4 Department of Obstetrics and Gynecology, University Hospital of Tarragona Joan XXIII. Institut d’Investigació Sanitària Pere Virgili (IISPV), Tarragona, Spain

**Correction to: Stem Cell Research & Therapy** 10.1186/s13287-021-02678-y

In the original article, the authors identified two editing errors: 

1. In the **Methods** section, concretely in the **Study population** subsection, a typing error was made, since it states that twenty-seven women were included in the study (14 with GDM and 14 with NGT), but indeed there are twenty-eight women.

2. In Table 1, the glycemia values are expressed in mmol/L but by error the units reflected in the table are mg/dL. The correct Table 1 has been provided here and the authors apologize for the inconveniences caused.

**Table 1:** Clinical and analytical characteristics of the cohort

	NGT (N = 14)	GDM (N = 14)	*P*-value
*Maternal clinical and analytical characteristics*			
Maternal age (years)	33.86 ± 6.97	36.57 ± 3.65	0.208
Pregestational BMI (kg/m^2^)	25.27 ± 3.29	30.33 ± 5.93	0.010
Gestational weight gain (kg)	12.37 ± 4.51	8.21 ± 6.10	0.051
Nulliparous, n (%)	6 (43)	2 (14)	0.092
Glucose (mmol/L)	4.88 ± 1.94	4.65 ± 1.03	0.704
*Offspring clinical and analytical characteristics*			
Gestational week delivery (weeks)	37.71 ± 0.73	38.07 ± 0.47	0.136
Birth weight (g)	3.265.36 ± 330.32	3.318.93 ± 379.89	0.694
Placental weight (g)	632.86 ± 47.51	675.00 ± 193.24	0.586
Sum skinfolds (mm)	12.47 ± 1.09	12.67 ± 2.20	0.773
Cb glucose (mmol/L)	3.57 ± 0.58	3.78 ± 0.78	0.437
Cb insulin mUI/L	33.89 ± 23.36	58.75 ± 50.83	0.295
Cb C peptide ng/dL	0.24 ± 0.06	0.27 ± 0.17	0.548

Data expressed as mean ± SD for quantitative variables following normal distribution, qualitative variables are expressed as n (%). Differences between quantitative variables were assessed by Student’s t-test or the Mann–Whitney U test, as required. Differences between qualitative variables were assessed by the chi-square test

*NGT* Normal Glucose Tolerance,* GDM* Gestational Diabetes Mellitus, *BMI* Body mass index, *Cb* Cord blood

